# Concomitant *TP53* Mutation Confers Worse Prognosis in *EGFR*-Mutated Non-Small Cell Lung Cancer Patients Treated with TKIs

**DOI:** 10.3390/jcm9041047

**Published:** 2020-04-07

**Authors:** Matteo Canale, Elisabetta Petracci, Angelo Delmonte, Giuseppe Bronte, Elisa Chiadini, Vienna Ludovini, Alessandra Dubini, Maximilian Papi, Sara Baglivo, Nicoletta De Luigi, Alberto Verlicchi, Rita Chiari, Lorenza Landi, Giulio Metro, Marco Angelo Burgio, Lucio Crinò, Paola Ulivi

**Affiliations:** 1 Biosciences Laboratory, Istituto Scientifico Romagnolo per lo Studio e la Cura dei Tumori (IRST) IRCCS, 47014 Meldola, Italy; matteo.canale@irst.emr.it (M.C.); elisa.chiadini@irst.emr.it (E.C.); 2 Biostatistics and Clinical Trials Unit, Istituto Scientifico Romagnolo per lo Studio e la Cura dei Tumori (IRST) IRCCS, 47014 Meldola, Italy; elisabetta.petracci@irst.emr.it; 3 Department of Medical Oncology, Istituto Scientifico Romagnolo per lo Studio e la Cura dei Tumori (IRST) IRCCS, 47014 Meldola, Italy; angelo.delmonte@irst.emr.it (A.D.); giuseppe.bronte@irst.emr.it (G.B.); alberto.verlicchi@irst.emr.it (A.V.); marco.burgio@irst.emr.it (M.A.B.); lucio.crino@irst.emr.it (L.C.); 4 Department of Medical Oncology, Santa Maria della Misericordia Hospital, 06129 Perugia, Italy; oncolab@hotmail.com (V.L.); baglivosara@gmail.com (S.B.); giulio.metro@yahoo.com (G.M.); 5 Department of Pathology, Morgagni-Pierantoni Hospital, 47121 Forlì, Italy; alessandra.dubini@auslromagna.it; 6 Department of Medical Oncology, Per gli Infermi Hospital, Rimini 47923, Italy; maximilianpapi@libero.it; 7 UOS Oncology, Istituto per la Sicurezza Sociale, State Hospital, Cailungo 47893, San Marino, Italy; nicolettadeluigi@hotmail.com; 8 Department of Medical Oncology, Ospedali Riuniti Padova Sud “M. Teresa di Calcutta”, ULSS6 Euganea, 35131 Padova, Italy; rita.chiari@aulss6.veneto.it; 9 Department of Medical Oncology, S. Maria delle Croci Hospital, 48121 Ravenna, Italy; landi.lorenza@gmail.com

**Keywords:** non-small-cell lung cancer, epidermal growth factor receptor, tyrosine kinase inhibitors, *TP53* mutations, responsiveness, prognosis

## Abstract

Background: Non-small cell lung cancer (NSCLC) is the primary cause of cancer-related deaths worldwide. Epidermal Growth Factor Receptor (*EGFR*)-mutated patients usually benefit from TKIs treatment, but a significant portion show unresponsiveness due to primary resistance mechanisms. We investigated the role of *TP53* mutations in predicting survival and response to *EGFR*-TKIs in EGFR-mutated NSCLC patients, to confirm, on an independent case series, our previous results. Methods: An independent retrospective cohort study was conducted, on a case series of 136 *EGFR*-mutated NSCLC patients receiving first or second generation TKIs as a first line therapy, and a smaller fraction of patients who acquired the T790M resistance mutation and were treated with third generation TKIs in the second or further line of treatment. *TP53* mutations were evaluated in relation to disease control rate (DCR), objective response rate (ORR), progression-free survival (PFS) and overall survival (OS) of the patients. Results: Forty-two patients (30.9%) showed a *TP53* mutation. Considered together, *TP53* mutations had no significant impact on time-to-event endpoints. Considering the different *TP53* mutations separately, exon 8 mutations confirmed their negative effect on PFS (HR 3.16, 95% 1.59–6.28, *p* = 0.001). In patients who developed the T790M resistance mutation, treated with third generation TKIs, the *TP53* exon 8 mutations predicted worse PFS (even though not statistically significant), and OS (HR 4.86, 95% CI: 1.25–18.90, *p* = 0.023). Conclusions: *TP53* exon 8 mutations confirmed their negative prognostic impact in patients treated with first and second generation TKIs and demonstrated a role in affecting clinical outcome in patients treated with third generation TKIs.

## 1. Introduction

Epidermal growth factor receptor (*EGFR*)-tyrosine kinase inhibitors (TKIs) have changed the natural history of non-small-cell lung cancer (NSCLC) patients harboring specific *EGFR* mutations at exons 18, 19 and 21. Randomized trials have demonstrated a median progression-free survival (PFS) of 9.7 and 9.5 months in patients harboring sensitizing *EGFR* mutations treated with first-generation *EGFR*-TKIs versus platinum-based chemotherapy [1,2], and 11.1 months for second generation TKIs [3]. Third generation TKI osimertinib, initially designed to overcome the arising of T790M resistance mutation in *EGFR* pre-treated patients [4], has recently become the gold standard for *EGFR*-mutated patients, reaching a median PFS of 18.9 months [5].

Despite the high sensitivity of *EGFR*-mutated patients to *EGFR*-TKIs, the objective response rate is of about 70–80% for 1st, 2nd and 3rd generation TKIs [2,3,5], meaning that a portion of patients do not respond to *EGFR*-TKI treatment, notwithstanding the presence of sensitizing *EGFR* mutation, suggesting the presence of primary resistance mechanisms.

Our previous study and several others showed that the concomitant presence of *TP53* mutation confers a worse prognosis in *EGFR*-mutated patients treated with first and second generation TKIs [6,7,8]. Subsequent studies performed using next generation sequencing methodologies showed that the presence of concomitant mutation in different genes is associated with a lower response to *EGFR-*TKIs and, however, *TP53* mutation confirms to be the most significant predictor of worse outcome. In particular, it seems that specific *TP53* mutations are more implicated in predicting the worse prognosis [6,9,10], confirming that different *TP53* mutations confer different p53 functions. Within the coding region of the *TP53* gene, several studies have reported that a higher frequency of mutations occurs in the exons 5–8, and that mutations in these exons are associated to differential functions of p53 protein [9,10]. As the different published studies have analyzed principally patients treated with first and second generation TKIs, few data are available with regard to the role of *TP53* mutation in relation to response to third generation TKIs.

The main purpose of this research was to confirm our previously published results on the role of *TP53* mutations, in an independent cohort of advanced *EGFR*-mutated patients treated with first or second generation TKIs in the first line setting, and to investigate the role of *TP53* mutations in predicting prognosis of patients with acquired T790M mutation treated with third generation TKIs.

## 2. Materials and Methods

To confirm our previous results on the role of *TP53* mutations in relation to the effectiveness of TKIs, an independent retrospective cohort study was conducted. All consecutive patients with advanced *EGFR*-mutated NSCLC receiving a first line TKI treatment (i.e., gefitinib, erlotinib, or afatinib) from July 2010 to May 2018 at the Medical Oncology Units of the Romagna catchment area (Area Vasta Romagna, AVR) and at the S. Maria della Misericordia Hospital of Perugia, Italy, were included in this study. Demographic and clinical characteristics of the patients were obtained using a medical and radiographic records review including age, gender, smoking history, histology, and information on death and response to treatment. *EGFR* status had been routinely determined at the Biosciences Laboratory of IRST-IRCCS and the Laboratory of Molecular Biology of the S. Maria della Misericordia Hospital, Perugia, by MassARRAY, pyrosequencing, direct sequencing or Next-Generation Sequencing (NGS) methodologies.

To evaluate the independent role of *TP53* mutations, that is, eventually adjusting for other covariates, and to obtain a more accurate estimate of their prognostic effect, an analysis combining the data of the present work with those from our previous one [6], was also performed, updating follow-ups of the previous case series to 30 June 2018.

Moreover, considering the two cohorts together, we identified a subgroup of 42 patients who developed the T790M resistance mutation and were treated with third generation TKI, osimertinib. All patients provided an informed consent, and the study was approved by the AVR Ethical Committee (study code IRST-B053).

### 2.1. EGFR and TP53 Mutation Analysis

*EGFR* mutation analyses were performed on both cytologic and histologic samples, accurately selected by a dedicated expert pathologist from each center at the time of diagnosis. The same DNA specimens were used for the determination of *TP53* mutation status, blindly to the clinical outcomes. Quality controls were periodically performed during the course of the study to ensure concordance of molecular results.

DNA was extracted by macro-dissection of an area comprising at least 50% of tumor cells. Cells were lysed in a digestion buffer of 50 mmol/L KCl, 10 mmol/L Tris-HCl pH 8.0, 2.5 mmol/L MgCl_2_, and Tween-20 0.45%; proteinase K at 1.25 mg/mL were added to each specimen, with an overnight incubation at 56 °C. After proteinase K inactivation at 95 °C for 10 min, samples were centrifuged twice to eliminate debris and supernatant DNA quantity and quality was assessed by Nanodrop (Celbio) before molecular analyses.

Mutation status for exons 5–8 of *TP53* gene was performed by PCR amplification and Direct Sequencing using 3130 Genetic Analyzer (Applied Biosystems, Monza, Italy), or Next-Generation Sequencing by Ion S5 platform (Thermofisher, Monza, Italy), or MySeq platform (Illumina, San Diego, CA, USA).

### 2.2. Response Evaluation

Best clinical response to treatment with TKI was classified on the basis of interval CT scans as complete response (CR), partial response (PR), stable disease (SD), or progressive disease (PD) using standard Response Evaluation Criteria in Solid Tumors criteria (RECIST) version 1.1. Patients with both baseline imaging and at least one repeated evaluation after continuous *EGFR*-TKI monotherapy were evaluable for radiographic response. The same criteria for response evaluation and periodicity were used by all centers taking part in the study.

### 2.3. Statistical Analyses

Data were summarized by mean ± standard deviation (SD) for continuous variables and through natural frequencies and percentages for categorical ones.

Treatment responses were reported as objective response rate (ORR) and disease control rate (DCR). The time-to-event endpoints examined were progression-free survival (PFS) and overall survival (OS). PFS was defined as the time from start of first line treatment (or from start of osimertinib for the subgroup analysis) to disease progression or death for any cause, whichever occurred first. Patients who were alive and progression-free at 30 June 2018, the last follow-up update, were censored at that date.

OS was defined as the time from start of first line treatment to death for any cause. Alive patients were censored at the date of the last follow-up update. PFS and OS functions were estimated using the Kaplan–Meier method, and the log-rank test was used to assess differences between groups. Median PFS and OS were reported as point estimates and 95% confidence intervals (CI) in round brackets. The Cox proportional hazards regression model was used to quantify the association between specific covariates and the time-to-event endpoints. Results are reported as HR and 95% CI in round brackets. To assess the association between mutations and the duration of response to TKIs, patients were divided into short-term responders (PFS less than 6 months), intermediate-term responders (PFS ≥6 months and ≤24 months) or long-term responders (PFS >24 months). The association between categorical variables was tested by the Pearson’s χ^2^ test or Fisher exact test, when appropriate, whereas those between a continuous variable and a categorical one was tested by means of the Student *t*-test or *F* test for more than two categories. To evaluate the independent role of *TP53* mutations in a multivariate analysis and to obtain more accurate estimates of their prognostic effect, a combined analysis including data of the present work with those from our previous one, was performed. Follow-up of our previous cohort was updated on 30 June 2018. A multivariable model was obtained using backward stepwise variable selection, setting the significance level for variable removal from the model equal to 0.10. In a perspective of parsimonious modelling, when appropriate, categories of some study variables were grouped. The proportional hazards assumption was evaluated using a statistical test based on Schoenfeld residuals. In case of non-proportional hazards for a specific variable, a Cox model with time-dependent coefficient, β(t), was fitted. To simplify model interpretation, a step function for β(t) was used, dividing the follow-up period in three time periods since treatment started: the first 6 months, 6–12 months, and greater than 12 months.

Overall and when not otherwise specified, a two-sided *p*-value (*p*) <0.05 was considered statistically significant. All statistical analyses were performed using STATA 15.0 software (College Station, TX, USA) and R version 3.6.1.

## 3. Results

### 3.1. Clinico-Pathologic and Molecular Features of Patients

Patients characteristics, *EGFR* mutations, type of TKI received and *TP53* mutations are reported in Table 1. All 136 patients carried an *EGFR* mutation (exon 19 deletions 53.7%, exon 21 L858R 35.3%, other *EGFR* mutations 11.0%) and received a first line *EGFR*-TKI (36.7% Erlotinib, 5.1% Erlotinib plus bevacizumab, 30.9% gefitinib, 27.2% afatinib). Of the patients with available information on smoking habit, half were never smokers (50.8%), and half were former or current smokers (28.8% and 20.3%, respectively). We found *TP53* mutations in 42 (30.9%) of the 136 analyzed patients: 12 mutations were in exon 5 (28.6%), 6 in exon 6 (14.3%), 13 in exon 7 (31.0%) and 11 in exon 8 (26.2%). Following the classification of *TP53* mutations into disruptive and non-disruptive ones [6], 11 patients had a disruptive mutation whereas 31 had a non-disruptive one (26.2% and 73.8%, respectively).

While for patients with exon 19 deletion the three different TKIs were used in an almost similar proportion (32.9% patients received erlotinib, 30.1% gefitinib, and 37.0% afatinib), patients with a mutation in exon 21 L858R were predominantly treated with erlotinib or gefitinib (52.1% and 39.6% of the patients, respectively), and those with uncommon mutations received predominantly erlotinib or afatinib (53.3% and 40.0%, respectively, Table S1).

No statistically significant associations were observed between type of *TP53* mutation, type of *EGFR* mutation and patient characteristics (Table S2).

### 3.2. Patients Outcome in Relation to EGFR Mutations

Overall, ORR and DCR were 67.4%, and 89.3%, respectively. Considering the clinical responses by type of *EGFR* mutation, ORR was considerably higher in the subgroup of patients with exon 19 deletion (77.5%), with respect to patients with L585R mutation (55.3%), and the subgroup with other mutations (54.6%), *p* = 0.029. A higher percentage of long responders was observed in patients carrying exon 19 deletion (12.3%), with respect to patients with L858R point mutation (7.3%), or patients with the other *EGFR* mutations (6.6%), Table 2.

Median PFS and OS were 12.3 (95% CI: 9.9–13.8) and 27.3 (95% CI: 21.9–52.9) months, respectively. No statistically significant association between PFS, OS and type of *EGFR* mutations was found (*p* = 0.282 and *p* = 0.207, respectively).

### 3.3. Patients Outcome in Relation to TP53 Mutations

**Figure 1:**
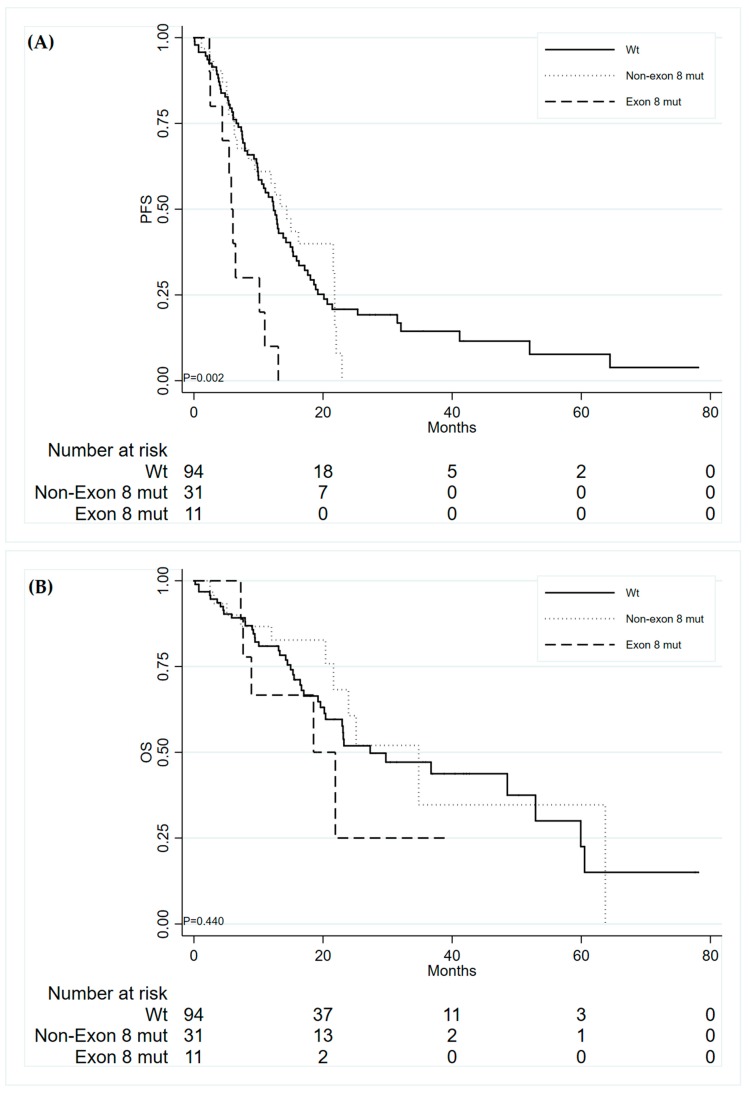
Progression-free survival (**A**) and Overall Survival (**B**) of patients according to *TP53.*

No statistically significant associations were found between *TP53* mutations and ORR and DCR (Table S3). When considering any type of *TP53* mutation with regard to PFS, no association was found; however, significant results were observed considering only *TP53* exon 8 mutations. As previously reported [6], patients with this gene mutation showed a shorter median PFS than non-exon 8 mutated and wild type *TP53* patients: 5.8 months (95% CI: 2.4–10.2) vs. 14.4 (95% CI: 6.7–21.8) and 12.4 (95% CI: 10.0–15.0), respectively (Figure 1A). These patients also showed a poorer OS as compared with the other groups, even though this result was not statistically significant: median OS were 18.53 months (95% CI: 7.3–NR), 34.8 (95% CI: 21.6–NR), 27.3 (95% CI: 20.2–52.9), respectively (Figure 1B).

The presence of *TP53* exon 8 mutation seemed to be associated with a worse prognosis in a similar way in the patients with the different *EGFR* mutations, both in terms of PFS and OS.

In particular, patients with wild type *TP53* exon 8 had a better clinical outcome independently by *EGFR* status: median PFS and OS were 12.9 (95% CI: 10.0–16.3) and 29.7 months (95% CI: 23.0–60.5) for patients with *EGFR* exon 19 deletion vs. 12.4 months (95% CI: 7.9–15.0) and 23.2 months (95% CI: 19.2–63.7) for those with other *EGFR* mutations, respectively; in the subgroup of patients with *TP53* exon 8 mutations, median PFS and OS were 5.8 months (95% CI: 2.5–NR) and 21.9 months (95% CI: 7.3–NR) for patients with *EGFR* exon 19 deletion vs. 6.4 (95% CI: 2.4–NR) and 18.5 months (95% CI: 7.6–NR) for those with other *EGFR* mutations. In Table S4, the univariate Cox analysis results are reported.

### 3.4. Multivariate Analysis of the Role of TP53 Mutation: Combined Cohorts of Patients

To obtain a more precise estimate of the effect of *TP53* exon 8 mutation on PFS and OS, and to determine its potential independent role considering other information, a pooled analysis considering either data from our previously analyzed cohort and the one described in the present study, was performed.

The final multivariate model for PFS included both *EGFR* exon 19 deletion as well as TP53 mutation. As soon as the effect of exon 19 deletion on the hazard of disease progression or death is not constant over time, that is, the proportional hazards assumption underlying the Cox model was violated, to obtain a better model fit, this variable was entered into the model with a time-dependent coefficient. Table 3 shows that the effect of exon 19 mutation changes over time, showing a strong protective effect over the first six months that vanishes afterward.

Adjusting for presence of *EGFR* exon 19 deletion, *TP53* mutations affecting exon 8 demonstrated to be the unique independent negative prognostic factor for PFS (HR 1.81, 95% CI: 1.13–2.92, Table 3). With regard to OS, only deletion in *EGFR* Exon 19 resulted associated to OS, probably due to data from our previous cohort (HR 0.52 (95% CI: 0.26–1.03) for the first 6 months of follow-up, HR 0.44 (95% CI: 0.22–0.90), for successive 6 months, and HR 1.08 (95% CI: 0.72–1.61) after 12 months).

### 3.5. TP53 Mutations in Relation to Responsiveness to Third Generation TKIs: Combined Cohorts of Patients

**Figure 2:**
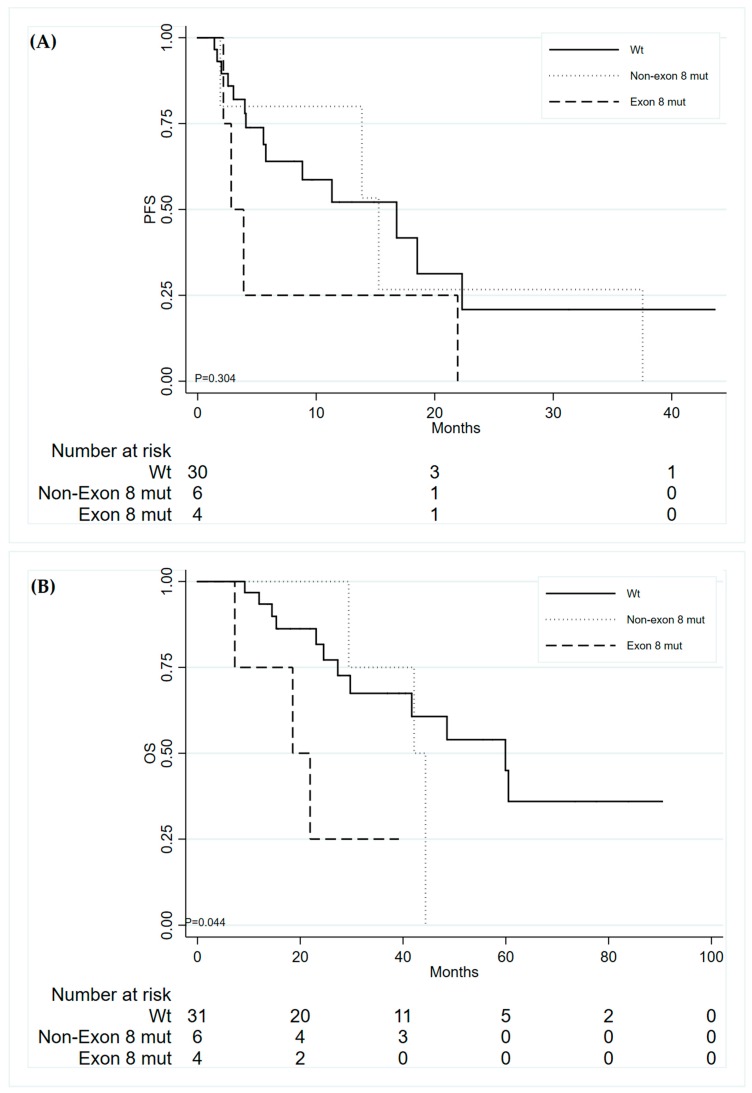
Progression-free survival (**A**) and Overall Survival (**B**) in relation to *TP53* mutations of patients with acquired T790M treated with third generation TKIs.

Considering both patients’ cohorts (*n* = 272 patients), we considered 42 patients who developed a T790M resistance mutation and were treated with third generation TKI osimertinib, in the second or further lines of therapy. Of these, 41 were evaluable for *TP53* mutation status; we found 10 *TP53* mutated patients (24.4%): 3 mutations in exon 5 (30%), 1 in exon 6 (10%), 2 in exon 7 (20%) and 4 in exon 8 (40%). Within the 41 patients with available clinical information, median PFS and OS were 13.86 (95% CI: 5.5–18.53) and 44.38 months (95% CI: 10.64–24.28), respectively. Median PFS of exon 8 *TP53* mutated patients was 2.83 (2.17–NR) months, with respect to a median PFS of 16.79 (5.55–22.31) and 15.28 (1.91–NR) months, for wt *TP53* and patients with mutations in other exons of the gene, respectively (Figure 2A). Even though a good separation, the difference among curves was not statistically significant (*p* = 0.304), due to small numbers of the exon 8 mutated patients. On the other hand, exon 8 *TP53* gene mutations significantly affected the survival of the patients, with a median OS for exon 8 *TP53* mutated patients of 18.53 (7.26–NR) months, with respect to 42.15 (29.43–NR) and 59.92 (29.73–NR) months of patients with mutations in other exons of *TP53* and wt *TP53*, respectively (*p* = 0.044) (Figure 2B). Table 4 shows the univariate hazard ratios for PFS and OS with respect to the presence of *TP53* exon 8 mutation.

## 4. Discussion

In this study, we analyzed *TP53* mutations in relation to clinical outcome in a large cohort of *EGFR*-mutated NSCLC patients receiving first or second generation TKIs as a first line therapy. Our results confirm that exon 8 *TP53* mutations are associated with a shorter PFS, in all settings of treatment.

Moreover, such a negative effect was also observed in the subgroup of patients treated with third generation after the development of T790M mutation.

Numerous studies demonstrated the role of *TP53* mutations in predicting poor prognosis of advanced NSCLC patients [9,11,12,13,14,15], and this was confirmed also in the subgroup of NSCLC patients carrying *EGFR* mutations [8,9,16]. In particular, different recent studies showed that the concurrent presence of *TP53* mutations negatively affects response to TKIs in *EGFR*-mutated NSCLC patients, suggesting a role for these gene mutations in determining primary resistance to these drugs [6,7,17,18,19,20]. *TP53* is the most frequently mutated gene in lung adenocarcinoma, with mutation rates reported up to 55% [13,21,22,23], with a predominantly clonal expression [24]. In our case series, we found 30% of patients carrying a *TP53* mutation in the exons 5–8, the same percentage we previously reported in an independent case series. It is well known that different *TP53* mutations lead to changes in the P53 protein that may have diverse biological significance [9,10,25], and mutations in the DNA-binding domain (exons 5–8), are frequently associated with gain-of-function properties, resulting in pro-oncogenic features of the P53 protein [26]. In our previous work, we found that exon 8 *TP53* mutations were able to predict worse response to *EGFR* TKIs, especially in the subgroup of patients with *EGFR* exon 19 deletion. In the present study we confirmed the negative prognostic value of *TP53* exon 8 mutation in an independent cohort of *EGFR*-mutated NSCLC treated with both first and second generation TKIs in the first line setting. In the present study, the prognostic value of exon 8 *TP53* mutation was evident independently from the type of *EGFR* mutation. In a combined analysis, we showed that the effect was evident on overall survival in *EGFR*-mutated patients who developed T790M at progression after first line TKIs and osimertinib.

These results, in agreement with those reported by Kim et al. [7], suggest a negative predictive role of *TP53* mutation in all lines of therapy and in relation to all TKIs. Furthermore, our results are consistent with a recent study that found that *TP53* mutations in exon 8 are associated with shorter OS of patients receiving a TKI as a first line treatment [27].

In another study, missense mutations in *TP53* gene resulted in shorter PFS in *EGFR* mutated patients treated with TKIs but showed no associations with PFS and OS in patients undergoing surgical resection [28]. According to Xu et al., who reported *TP53* mutations in 88% of NSCLC *EGFR*-mutated patients that responded for <6 months to an *EGFR* TKIs, with respect to 13% of responders for >24 months [29], our results show a higher rate of *TP53* mutations in non-responders group, with no *TP53* mutated patients in the long responder group.

To investigate the role of *TP53* mutations in predicting clinical outcome of patients treated with third generation TKIs, we considered 42 patients’ developed T790M mutation to first line treatment with first or second generation TKI and received a third generation drug in the second or further line of therapy. In this subgroup, we found a diminished PFS in patients carrying *TP53* mutations in exon 8, even though without statistical significance, probably due to the small number of analyzed patients; exon 8 *TP53* mutated patients had a significantly shorter OS, with respect to wt *TP53* patients and patients with mutations in other exons of *TP53*. This observation is consistent with previous observations, that identified *TP53* mutations (not only in exon 8) as a negative prognostic predictor [7,16]. This result was not confirmed by a study from Labbé et al., that found no differences in ORR of patients treated with third generation TKIs, based on *TP53* mutation status; this could be for the small size of the analyzed case series [28]. In the light of the paradigm shift brought by FLAURA trial [5], there is a need to identify which biomarkers could predict primary resistance to osimertinib as a first line therapy; if confirmed in a larger case series treated with third generation TKI in the first line, these results could help to better stratify patients, suggesting an *EGFR*-independent mechanism of resistance, as others have already highlighted [30].

## 5. Conclusions

In conclusion, we confirmed that *TP53* exon 8 mutations identify a subgroup of patients with primary resistance to *EGFR* TKIs, and that this is true also in relation to third generation TKIs such as osimertinib. These data suggest that patients with concomitant *EGFR* and exon 8 *TP53* mutations should be candidates for more aggressive therapeutic schemes and should be monitored with a stricter follow-up.

## Figures and Tables

**Table 1:** Demographic, clinicopathological and molecular characteristics of patients (*n* = 136).

Characteristic	*n*	(%)
**Gender**		
Female	86	(62.2)
Male	50	(36.8)
**Age at first line TKI**		
Mean ± SD	67.6 ± 11.2	
**Smoking habit ^†^**		
Never smoker	60	(50.9)
Former smoker	34	(28.8)
Current smoker	24	(20.3)
**Type of *EGFR* mutation**		
Exon 19 deletion	73	(53.7)
Exon 21 L858R	48	(35.3)
Other uncommon mutations	15	(11.0)
**Type of *EGFR* exon 19 deletion**		
No exon 19 deletion	63	(46.3)
Deletion starts at codon 746	62	(45.6)
Deletion starts at codon 747	11	(8.1)
**Type of TKI received in first line setting**		
Erlotinib *	57	(41.9)
Gefitinib	42	(30.9)
Afatinib	37	(27.2)
***TP53* mutation**		
Wild type	94	(69.1)
Exon 5	12	(8.8)
Exon 6	6	(4.4)
Exon 7	13	(9.6)
Exon 8	11	(8.1)
**Type of *TP53* mutation**		
Wild type	94	(69.1)
Disruptive	11	(8.1)
Non-disruptive	31	(22.8)

**^†^** The sum does not add up to the total due to missing values. * Of these patients, 7 received Erlotinib plus Bevacizumab as a first line therapy, as provided in the Beverly clinical trial.

**Table 2:** Best clinical response according to *EGFR* mutations.

	All *EGFR* Mutations (*n* = 136)		Exon 19 Deletion (*n* = 73)		Exon 21 L858R (*n* = 48)		Other *EGFR* Mutations (*n* = 15)		*p*
	*n*	(%)	*n*	(%)	*n*	(%)	*n*	(%)	
**Best response ^†^**									0.026
**CR**	13	(9.9)	8	(11.3)	4	(8.5)	1	(7.1)	
**PR**	76	(57.6)	47	(66.2)	22	(46.8)	7	(50.0)	
**SD**	29	(22.0)	7	(9.9)	17	(36.2)	5	(35.7)	
**PD**	14	(10.6)	9	(12.7)	4	(8.5)	1	(7.1)	
**ORR**	89	(67.4)	55	(77.5)	26	(55.3)	6	(54.6)	0.029
**DCR**	118	(89.4)	62	(87.3)	43	(91.5)	11	(100.0)	0.844
**Duration of response**									0.260
**Short-term responders**	36	(26.4)	19	(26.0)	10	(20.8)	7	(46.7)	
**Medium-term responders**	87	(64.0)	45	(61.6)	35	(72.9)	7	(46.7)	
**Long-term responders**	13	(9.6)	9	(12.3)	2	(6.3)	1	(6.6)	

**^†^** The sum does not add up to the total due to missing values.

**Table 3:** Multivariate Cox analysis of progression-free survival (PFS) (*n* = 272).

	PFS		
	HR	95% CI	*p*
**Exon 19 deletion**			
No	1		
Yes			
0–6 months	0.56	(0.35–0.89)	0.014
6–12 months	0.67	(0.40–1.12)	0.123
>12 months	1.27	(0.80–2.03)	0.314
***TP53* exon 8 mutations**			
Wild-type *TP53*	1		
Non-Exon 8 mutations	1.02	0.73–1.42	0.905
Exon 8 mutations	1.81	1.13–2.92	0.014

**Table 4:** *TP53* mutations in relation to progression-free survival (PFS) and overall survival (OS) of patients receiving osimertinib in second or further lines of treatment.

	PFS			OS		
	HR	(95% CI)	*p*	HR	(95% CI)	*p*
*TP53* Exon 8						
Wild type	1			1		
Non-Exon 8 mutations	1.15	(0.37–3.59)	0.811	1.55	(0.42–5.76)	0.514
Exon 8 mutations	2.39	(0.77–7.45)	0.134	4.86	(1.25–18.90)	0.023

## Supplementary Materials

The following are available online at https://www.mdpi.com/2077-0383/9/4/1047/s1, Table S1: Demographic, clinicopathological and molecular characteristics of patients according to *EGFR* mutations (*n* = 136); Table S2: Demographic, clinicopathological and molecular characteristics by *TP53* mutation; Table S3: Best clinical response according to *TP53* mutations; Table S4: Univariate Cox analyses for PFS and OS.

## References

[1] Rosell R., Carcereny E., Gervais R., Vergnenègre A., Massuti B., Felip E., Palmero R., Garcia-Gomez R., Pallares C., Sanchez J.M. (2012). Erlotinib versus standard chemotherapy as first-line treatment for European patients with advanced EGFR mutation-positive non-small-cell lung cancer (EURTAC): a multicentre, open-label, randomised phase 3 trial. Lancet Oncol..

[2] Mok T.S., Wu Y.-L., Thongprasert S., Yang J.C.-H., Chu D.-T., Saijo N., Sunpaweravong P., Han B., Margono B., Ichinose Y. (2009). Gefitinib or Carboplatin–Paclitaxel in Pulmonary Adenocarcinoma. New Engl. J. Med..

[3] Yang J.C.-H., Wu Y.-L., Schuler M., Sebastian M., Popat S., Yamamoto N., Zhou C., Hu C.-P., O’Byrne K., Feng J. (2015). Afatinib versus cisplatin-based chemotherapy for EGFR mutation-positive lung adenocarcinoma (LUX-Lung 3 and LUX-Lung 6): analysis of overall survival data from two randomised, phase 3 trials. Lancet Oncol..

[4] Mok T.S., Wu Y.-L., Ahn M.-J., Garassino M.C., Kim H.R., Ramalingam S.R., Shepherd F.A., He Y., Akamatsu H., Theelen W.S. (2016). Osimertinib or Platinum-Pemetrexed in EGFR T790M-Positive Lung Cancer. New Engl. J. Med..

[5] Soria J.-C., Ohe Y., Vansteenkiste J., Reungwetwattana T., Chewaskulyong B., Lee K.H., Dechaphunkul A., Imamura F., Nogami N., Kurata T. (2018). Osimertinib in UntreatedEGFR-Mutated Advanced Non–Small-Cell Lung Cancer. New Engl. J. Med..

[6] Canale M., Petracci E., Delmonte A., Chiadini E., Dazzi C., Papi M., Capelli L., Casanova C., De Luigi N., Mariotti M. (2016). Impact of TP53 Mutations on Outcome in EGFR-Mutated Patients Treated with First-Line Tyrosine Kinase Inhibitors. Clin. Cancer Res..

[7] Kim Y., Lee B., Shim J.H., Lee S.-H., Park W.-Y., Choi Y.-L., Sun J.-M., Ahn J.S., Ahn M.-J., Park K. (2019). Concurrent Genetic Alterations Predict the Progression to Target Therapy in EGFR-Mutated Advanced NSCLC. J. Thorac. Oncol..

[8] VanderLaan P.A., Rangachari D., Mockus S.M., Spotlow V., Reddi H.V., Malcolm J., Costa D.B. (2018). Mutations in TP53, PIK3CA, PTEN and other genes in EGFR mutated lung cancers: Correlation with clinical outcomes. Lung Cancer.

[9] Molina-Vila M.A., Bertran-Alamillo J., Gascó A., Mayo-de-las-Casas C., Sánchez-Ronco M., Pujantell-Pastor L., Majem M. (2014). Nondisruptive p53 Mutations Are Associated with Shorter Survival in Patients with Advanced Non–Small Cell Lung Cancer. Clin. Cancer Res..

[10] Poeta M.L., Manola J., Goldwasser M.A., Forastiere A., Benoit N., Califano J.A., Ridge J.A., Goodwin J., Kenady D., Saunders J. (2007). TP53 mutations and survival in squamous-cell carcinoma of the head and neck. N. Engl. J. Med..

[11] La Fleur L., Falk-Sörqvist E., Smeds P., Berglund A., Sundström M., Mattsson J.S., Brandén E., Koyi H., Isaksson J., Brunnström H. (2019). Mutation patterns in a population-based non-small cell lung cancer cohort and prognostic impact of concomitant mutations in KRAS and TP53 or STK11. Lung Cancer.

[12] Zhao J., Han Y., Li J., Chai R., Bai C. (2019). Prognostic value of KRAS/TP53/PIK3CA in non-small cell lung cancer. Oncol Lett..

[13] Volckmar A.-L., Leichsenring J., Kirchner M., Christopoulos P., Neumann O., Budczies J., De Oliveira C.M.M., Rempel E., Buchhalter I., Brandt R. (2019). Combined targeted DNA and RNA sequencing of advanced NSCLC in routine molecular diagnostics: Analysis of the first 3,000 Heidelberg cases. Int. J. Cancer.

[14] Jiao X.-D., Qin B.-D., You P., Cai J., Zang Y.-S. (2018). The prognostic value of TP53 and its correlation with EGFR mutation in advanced non-small cell lung cancer, an analysis based on cBioPortal data base. Lung Cancer.

[15] Gu J., Zhou Y., Huang L., Ou W., Wu J., Li S., Xu J., Feng J., Liu B. (2016). TP53 mutation is associated with a poor clinical outcome for non-small cell lung cancer: Evidence from a meta-analysis. Mol. Clin. Oncol..

[16] Aggarwal C., Davis C.W., Mick R., Thompson J.C., Ahmed S., Jeffries S., Bagley S., Gabriel P., Evans T.L., Bauml J.M. (2018). Influence of TP53 Mutation on Survival in Patients With Advanced EGFR-Mutant Non–Small-Cell Lung Cancer. JCO Precis. Oncol..

[17] Yu H.A., Arcila M.E., Rekhtman N., Sima C.S., Zakowski M.F., Pao W., Kris M., Miller V.A., Ladanyi M., Riely G.J. (2013). Analysis of tumor specimens at the time of acquired resistance to EGFR-TKI therapy in 155 patients with EGFR-mutant lung cancers. Clin. Cancer Res..

[18] Hou H., Qin K., Liang Y., Zhang C., Liu N., Jiang H., Liu K., Zhu J., Lv H., Li T. (2019). Concurrent TP53 mutations predict poor outcomes of EGFR-TKI treatments in Chinese patients with advanced NSCLC. Cancer Manag. Res..

[19] Jin Y., Shi X., Zhao J., He Q., Chen M., Yan J., Ou Q., Wu X., Shao Y.W., Yu X. (2018). Mechanisms of primary resistance to EGFR targeted therapy in advanced lung adenocarcinomas. Lung Cancer.

[20] Chen M., Xu Y., Zhao J., Zhong W., Zhang L., Bi Y., Wang M. (2019). Concurrent Driver Gene Mutations as Negative Predictive Factors in Epidermal Growth Factor Receptor-Positive Non-Small Cell Lung Cancer. EBioMedicine.

[21] (2014). Comprehensive molecular characterization of gastric adenocarcinoma. Nature.

[22] (2014). Corrigendum: Comprehensive molecular profiling of lung adenocarcinoma. Nature.

[23] Shi J., Hua X., Zhu B., Ravichandran S., Wang M., Nguyen C., Brodie S.A., Palleschi A., Alloisio M., Pariscenti G. (2016). Somatic Genomics and Clinical Features of Lung Adenocarcinoma: A Retrospective Study. PLoS Med..

[24] Jamal-Hanjani M., Wilson G.A., McGranahan N., Birkbak N.J., Watkins T.B., Veeriah S., Shafi S., Johnson D.H., Mitter R., Rosenthal R. (2017). Tracking the Evolution of Non-Small-Cell Lung Cancer. New Engl. J. Med..

[25] Brosh R., Rotter V. (2009). When mutants gain new powers: news from the mutant p53 field. Nat. Rev. Cancer.

[26] Muller P.A.J., Vousden K.H. (2013). P53 mutations in cancer. Nat. Cell Biol..

[27] Liu Y., Xu F., Wang Y., Wu Q., Wang B., Yao Y., Zhang Y., Han-Zhang H., Ye J., Zhang L. (2019). Mutations in exon 8 of TP53 are associated with shorter survival in patients with advanced lung cancer. Oncol. Lett..

[28] Labbé C., Cabanero M., Korpanty G.J., Tomasini P., Doherty M.K., Mascaux C., Leighl N.B. (2017). Lung Cancer Prognostic and predictive e ff ects of TP53 co-mutation in patients with EGFR-mutated non-small cell lung cancer (NSCLC). Lung Cancer.

[29] Xu Y., Tong X., Yan J., Wu X., Shao Y.W., Fan Y. (2018). Short-Term Responders of Non–Small Cell Lung Cancer Patients to EGFR Tyrosine Kinase Inhibitors Display High Prevalence of TP53 Mutations and Primary Resistance Mechanisms. Transl. Oncol..

[30] Leonetti A., Sharma S., Minari R., Perego P., Giovannetti E., Tiseo M. (2019). Resistance mechanisms to osimertinib in EGFR-mutated non-small cell lung cancer. Br. J. Cancer.

