# Supplementary material for: Concomitant TP53 Mutation Confers Worse Prognosis in EGFR-Mutated Non-Small Cell Lung Cancer Patients Treated with TKIs
Source: J Clin Med. 2020 Apr 7;9(4):1047. doi: 10.3390/jcm9041047 (PMC7230306; doi:10.3390/jcm9041047)
Supplement: Supplementary file 1 [file jcm-09-01047-s001.pdf]

Table S1. Demographic, clinicopathological and molecular characteristics of patients according to EGFR mutations (n = 136).

	**Ex 19 del (n=73)**		**Ex 21 L858R (n=48)**		**Other EGFR mutations (n=15)**		**P-value**
	n	(%)	n	(%)	n	(%)	
**Characteristic**							
**Gender**							0.948
F	46	63.0	31	64.6	9	60.0	
M	27	37.0	17	35.4	6	40.0	
**Age at first-line TKI**							
median ± SD	65.5 ± 11.4		70.8 ± 10.4		67.9 ± 11.3		**0.037**
Missing							
**Smoking habit**							0.090
Never smoker	34	56.7	21	48.8	5	33.3	
Former smoker	15	25.0	16	37.2	3	20.0	
Current smoker	11	18.3	6	14.0	7	46.7	
Missing							
**Type of TKI received**							**0.001**
Erlotinib/ Erlotinib + Bevacizumab	24	32.9	25	52.1	8	53.3	
Gefitinib	22	30.1	19	39.6	1	6.7	
Afatinib	27	37.0	4	8.3	6	40.0	

Table S2. Demographic, clinicopathological and molecular characteristics by *TP53* mutation

	***TP53* mutation**		
	**Wt** n (%)	**Mut** n (%)	**P**
**Characteristic**			
**Gender**			0.865
F	59 (62.8)	27 (64.3)	
M	35 (37.2)	15 (35.7)	
**Age at first-line TKI**			0.361
Mean ± SD	68.2 ± 11.02	66.3 ± 1.8	
**Smoking habit**			0.424
Never smoker	44 (55.0)	16 (42.1)	
Former smoker	21 (26.3)	13 (34.2)	
Current smoker	15 (18.8)	9 (23.7)	
**Type of EGFR mut**			0.400
Exon 19 deletion	54 (57.5)	19 (45.2)	
Exon 21 L858R	30 (31.9)	18 (42.9)	
Exon 18/exon21 L861Q/other uncommon mut	10 (10.6)	5 (11.9)	
**Type of TKI received in first line**			0.918
Erlotinib/Erlotinib+Bevacuzumab	40 (42.6)	17 (40.5)	
Gefitinib	28 (29.8)	14 (33.3)	
Afatinib	26 (27.7)	11 (26.2)	

Table S3. Best clinical response according to *TP53* mutations

	ORR				P	DCR				P
	No (n=43)		Yes (n=89)			No (n=14)		Yes (n=118)		
	n	(%)	n	(%)		n	(%)	n	(%)	
**All mutations**					0.586					1.000
Wt	31	72.09	60	67.42		10	71.43	81	68.64	
Mut	12	27.91	29	32.58		4	28.57	37	31.36	
**Exon 5**					0.476					1.000
Wt	31	72.09	60	67.42		10	71.43	81	68.64	
Non-exon 5 mut	7	16.28	22	24.72		3	21.43	26	22.03	
Exon 5 mut	5	11.63	7	7.87		1	7.14	11	9.32	
**Exon 6**					0.274					1.000
Wt	31	72.09	60	67.42		10	71.43	81	68.64	
Non-exon 6 mut	12	27.91	23	25.84		4	28.57	31	26.27	
Exon 6 mut	0	0.00	6	6.74		0	0.00	6	5.08	
**Exon 7**					0.915					1.000
Wt	31	72.09	60	67.42		10	71.43	81	68.64	
Non-exon 7 mut	8	18.60	20	22.47		3	21.43	25	21.19	
Exon 7 mut	4	9.30	9	10.11		1	7.14	12	10.17	
**Exon 8**					0.908					0.408
Wt	31	72.09	60	67.42		10	71.43	81	68.64	
Non-exon 8 mut	9	20.93	22	24.72		2	14.29	29	24.58	
Exon 8 mut	3	6.98	7	7.87		2	14.29	8	6.78	
**Disruptive/ nondisruptive**					0.432					1.000
Wt/disruptive	35	81.40	67	75.28		11	78.57	91	77.12	
Nondisruptive	8	18.60	22	24.72		3	21.43	27	22.88	

Table S4. Univariate Cox analyses for PFS and OS

		PFS			OS	
	**HR**	**(95% CI)**	**P**	**HR**	**(95% CI)**	**P**
**Gender**						
Female	1			1		
Male	1.13	(0.76 – 1.69)	0.546	1.64	(0.97 – 2.77)	0.066
**Age at first-line TKI**	1.00	(0.98 – 1.01)	0.872	1.03	(1.00 – 1.05)	0.034
**Smoking habit**						
Never smoker	1			1		
Former smoker	1.17	(0.73 – 1.88)	0.510	1.06	(0.56 – 2.01)	0.849
Current smoker	1.03	(0.58 – 1.84)	0.911	0.99	(0.44 – 2.21)	0.980
**Type of EGFR mutation**						
Other mutations	1			1		
Exon 19 deletion	0.93	(0.63 – 1.37)	0.714	0.87	(0.51 – 1.44)	0.560
**Type of TKI received in first-line setting**						
Erlotinib*	1			1		
Gefitinib	0.75	(0.47 – 1.19)	0.225	0.90	(0.84 – 1.69)	0.746
Afatinib	0.79	(0.49 – 1.27)	0.324	0.99	(0.52 – 1.90)	0.988
**Any *TP53* mutation**						
Wt	1			1		
Mutated	1.22	(0.80 – 1.86)	0.356	0.96	(0.54 – 1.72)	0.898
***TP53* Exon 5**						
Wt	1			1		
Non-Exon 5 mutations	1.23	(0.76 – 1.99)	0.390	1.04	(0.55 – 1.98)	0.907
Exon 5 mutations	1.19	(0.60 – 2.32)	0.611	0.79	(0.28 – 2.21)	0.651
***TP53* Exon 6**						
Wt	1			1		
Non-Exon 6 mutations	1.34	(0.87 – 2.06)	0.189	1.14	(0.63 – 2.08)	0.669
Exon 6 mutations	0.64	(0.20 – 2.05)	0.457	0.29	(0.04 – 2.19)	0.233
***TP53* Exon 7**						
Wt	1			1		
Non-Exon 7 mutations	1.41	(0.87 – 2.27)	0.161	0.88	(0.44 – 1.75)	0.712
Exon 7 mutations	0.93	(0.48 – 1.82)	0.840	1.15	(0.49 – 2.72)	0.751
***TP53* Exon 8**						
Wt	1			1		
Non-Exon 8 mutations	0.96	(0.60 – 1.55)	0.878	0.81	(0.42 – 1.58)	0.545
Exon 8 mutations	3.16	(1.59 – 6.28)	0.001	1.62	(0.63 – 4.13)	0.313
**Type of *TP53* mutations**						
Wt	1			1		
Disruptive mutations	0.89	(0.43 – 1.85)	0.752	0.54	(0.17 – 1.75)	0.302
Non-disruptive mutations	1.38	(0.87 – 2.20)	0.169	1.18	(0.63 – 2.22)	0.604

* Of these patients, 7 received Erlotinib plus Bevacizumab as a first-line therapy, as provided in the Beverly clinical trial
